# Soil Moisture and Available Phosphorus as the Factors Driving Variation in Functional Characteristics across Different Restoration Communities in a Subtropical Mountain Ecosystem

**DOI:** 10.3390/biology12030427

**Published:** 2023-03-10

**Authors:** Xiaoni Wu, Chunjie Shen, Xudong Ma, Lianyu Hu, Yongjian He, Huaye Shang, Denggao Fu

**Affiliations:** 1 School of Agronomy and Life Sciences, Kunming University, Kunming 650214, China; wuxiaoxiaoni@163.com; 2 Yunnan Key Laboratory for Plateau Mountain Ecology and Restoration of Degraded Environments, School of Ecology and Environmental Sciences, Yunnan University, Kunming 650091, China; ecozhaoluo@163.com (C.S.); ecolibo@163.com (X.M.); ecohuang11@163.com (L.H.); ecoforest2023@126.com (Y.H.); shanghuaye28@126.com (H.S.); 3 Yunnan International Cooperative Center of Plateau Lake Ecological Restoration and Watershed Management & Yunnan Think Tank of Ecological Civilization, Kunming 650091, China

**Keywords:** functional traits, functional diversity, community-weighted mean, species diversity, distribution pattern, habitat filter

## Abstract

**Simple Summary:**

Successful restoration requires evaluating the patterns and driving factors of functional characteristics under different land restoration types. The functional properties of communities are frequently quantified using functional diversity (FD) and the community-weighted mean (CWM). Changes in functional characteristics in different restoration communities are influenced not only by species attributes but also by site environmental conditions. However, the patterns and driving factors of functional characteristics have been poorly explored. Here, we investigated species diversity, functional characteristics, and soil physicochemical properties across four vegetation restoration types. We found that different restoration communities significantly altered most community structures and functional properties in terms of species diversity, FD, and CWM. Most of the variation in functional characteristics was explained by soil physicochemical properties, especially SWC and AP. Moreover, the linkages between CWMs and soil properties were stronger than those between FD and soil properties. These results indicated that SWC and AP, as habitat filters, regulated the patterns of functional characteristics by changing the dominant species and their functional traits.

**Abstract:**

Functional characteristics are increasingly used to evaluate the success of different vegetation restoration. Community functional diversity (FD) and the community-weighted mean (CWM), as two main complementary components, are closely linked to site environment and ecosystem functions. However, the patterns and driving factors of functional characteristics are still not clear in different vegetation restoration types. Here, four community restoration types (secondary shrubland, SL; *Pinus yunnanensis* forest, PF; mixed needle–broad-leaved forest, MF; natural secondary forest, NSF) were selected to investigate species diversity, FD, CWM, and soil physicochemical properties. The relative effects of species diversity and soil abiotic features on variation in functional characteristics were then evaluated. We found that different restoration communities altered most community structures and functional properties in terms of species diversity, FD, and CWM. CWM values and FD in different communities presented different distribution patterns depending on certain traits and parameters. Significant correlations between functional traits were found at the species and community scales, suggesting a potential covariation between these selected traits in communities. The results of redundancy analysis and variation partitioning showed that most of the variation in functional characteristics, especially CWM, was explained by soil moisture and available phosphorus, indicating that habitat filters regulate the functional characteristics of plant communities mainly by changing the dominant species composition and functional traits of species. Therefore, the selection of restoration species adapted to low soil moisture and available phosphorus and the construction of communities based on selected species as the dominant species can effectively drive community assembly and ecosystem functions in the vegetation restoration process.

## 1. Introduction

Biodiversity is a key element of vegetation restoration and has been recognized as an important criterion of successful restoration [1,2,3]. Species diversity patterns and effects on ecosystem processes and functions under different restoration communities have been widely reported in different climatic and land-use types [4,5]. While taxonomic studies typically describe species composition changes, functional characteristics at the species and community levels are receiving more attention in many research works, exploring the relationships among environment, functional traits, and ecosystem functions [6,7,8] and revealing the mechanisms behind the consequences of diversity changes on ecological processes [9]. Therefore, it is necessary to evaluate the patterns of functional characteristics under different vegetation restoration types.

At the community level, the functional properties of communities are frequently quantified using functional diversity (FD) and the community-weighted mean (CWM). FD represents the distribution pattern of species in trait space [10], which mainly includes functional richness (FRic), functional evenness (FEve), functional division (FDiv), and functional dispersion (FDis). FRic reflects the functional space occupied by a species. FEve implies the evenness of the distribution of functional traits of community species and reflects the effective utilization efficiency of resources. The values of FDiv and FDis indicate the degree of complementarity of niche and resource competition between species in a community. The CWM of functional traits demonstrates the dominant trait in a community [11]. According to the mass ratio hypothesis, CWMs and ecological processes are tightly related [12]. Thus, functional characteristics and species diversity are considered as important diversity parameters indicating community structural and functional attributes. Many studies have reported differences in species diversity and functional characteristics (FD and CWM) between restoration vegetation in different climatic and land-use types [13,14,15]. However, the pattern of functional characteristics and the relationship between species and functional characteristics remain unclear in different restoration community types and thus have strong implications for ecological restoration and management.

Changes in functional characteristics in different plant communities are influenced not only by species attributes such as biological characteristics and species diversity [16,17,18] but also by the site environment such as the soil carbon and nutrients [19,20]. The general idea is that a higher species diversity is often associated with a higher FD because species diversity can increase the functional differences among species [17]. Thus, community functional characteristics are largely affected by the composition and distribution of species. However, the existence of species does not necessarily display the functional distinction among species [21]. In addition, some research has reported that functional traits and FD are linked with the abiotic environment, such as the availability of light [19,22]. Meanwhile, soil physicochemical properties can cause a change in community species diversity, and thus affect patterns of community functional characteristics. For example, topographic position has an important role in regulating functional strategies in tropical dry forest trees by changing light, water and nutrients conditions [23,24]. However, we know little about soil properties and their relation to functional attributes in different plant communities. Moreover, the relative effects of soil properties and species diversity on the functional characteristics of plant communities have been poorly explored.

In the present study, different vegetation restoration types, including secondary shrubland (SL), *Pinus yunnanensis* forest (PF), mixed needle–broad-leaved forest (MF), and natural secondary forest (NSF), were selected for investigation of their community attributes and soil abiotic properties. Our aims were (i) to analyze the distribution patterns of species diversity, FD, and CWM in different vegetation restoration types, (ii) to quantify the correlations among functional traits and between species diversity and functional characteristics, and (iii) to evaluate the relative effects of species diversity and soil resources on community functional characteristics. We hypothesized that changes in soil resource conditions under different restoration communities would influence species composition and abundance as habitat filters, and thus regulate the functional characteristics of plant communities. 

## 2. Materials and Methods

### 2.1. Study Area

The study area is located at the ecological observation station of Mouding County (25°24′09″ N; 101°28′18″ E), Yunnan Province, China. The area has an average annual rainfall of 846 mm and a temperature of 16 °C. Every year, the rainy season lasts from May to October. The soil of the area is reddish. A subtropical evergreen broad-leaved forest made up the majority of the original vegetation, which was coppiced for use as pastures or as fuelwood. The majority of the degraded areas have been planted with the quickly expanding *P. yunnanensis* since the 1980s. After a second natural succession, other remnant coppices and pastures developed distinct plant community types. Here, four main vegetation restoration types in the area, including secondary shrubland (SL), *P. yunnanensis* coniferous forest (PF), mixed needle–broad-leaved forest (MF), and natural secondary forest (NSF), were selected to investigate community structural attributes, species functional traits, and soil properties.

### 2.2. Community Composition Investigation and Sampling Analysis

For each plant community type, five duplicate plots were established. The distance among the plots in each community type was more than 200 m. All 12 plots shared the same climatic and soil zones, as well as a similar slope direction (20–25° west of north) and gradient (15–17°). We began by identifying all of the woody species and measuring the heights and diameters at breast height (DBH) of all woody plants ≥ 1.3 m height. Saplings and woody seedlings (<1.3 m) were also identified, and abundance was estimated using the basal area or percentage cover. According to this scheme, the species relative abundance and species diversity were calculated. Second, we evaluated five representative traits: specific leaf area (SLA), leaf dry matter content (LDMC), leaf nitrogen and phosphorus concentrations (LNC and LPC), and specific root length (SRL) [25]. For each species, these traits were measured on at least ten random individuals. The traits of a total of 27 common species were measured following standardized protocols [25]. FRic, FEve, FDiv, and FDis were used to indicate functional diversity [10]. With the use of species trait values and species abundance, CWMs were generated for each trait. The FDiversity software tool was used to calculate the species diversity, as well as all of the indicators of community functional characteristics [26]. In addition, 5 random soil cores (0–30 cm) were sampled in each plot. They were pooled, sieved, and analyzed for basic soil properties, including soil pH, soil bulk density (BD), soil water content (SWC), soil organic matter (SOM), total nitrogen (TN) and available nitrogen (AN), and total phosphorus (TP) and available phosphorus (AP) [27].

### 2.3. Statistical Analysis 

Species diversity, FD, CWM, and soil physical and chemical properties among four community restoration types were first compared by one-way ANOVA. Second, Spearman correlations were used to analyze the relationships between functional traits at the species level and CWM at the community level. Third, simple correlation analysis and redundancy analysis (RDA) were performed to quantify the correlations among soil properties, species diversity, FD, and CWM. Moreover, we analyzed the linkages between biplot scores from RDA and influencing factors using the function *envfit* and permutation tests. Finally, to quantify the relative effects of species diversity and soil abiotic properties for the FD in the four communities, variance partitioning analysis was performed to illustrate the explanatory power (anova.cca function in the *vegan* package) using R [28].

## 3. Results

### 3.1. Community Structural Properties and Soil Properties

**Figure 1:**
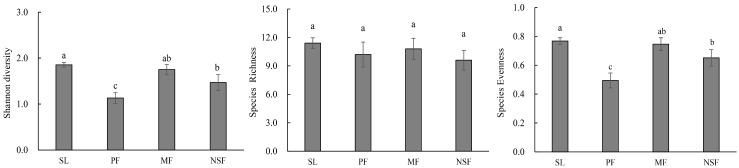
Species diversity (Shannon diversity, species richness, and species evenness) in the four vegetation restoration types. The error bars represent the standard deviation of the mean. Different letters represent significant differences at the 0.05 level.

In terms of species composition, shrubland was dominated by *Eupatorium adenophorum*, *Ternstroemia gymnanthera*, and *Vaccinium fragile*. *P. yunnanensis* and *Imperata cylindrica* were the dominant species in PF. *P. yunnanensis*, *Keteleeria evelyniana*, and *Cyclobalanopsis glaucoides* were the dominant trees in MF. *K. evelyniana*, *C. glaucoides*, and *Lithocarpus dealbatus* were dominant in NSF. The average cover and the stand density of the dominant plants in the tree layer were the highest in NSF (Table 1). There were significant differences (*p* < 0.05) in the Shannon diversity index and species evenness among SL, MF, and NSF (Figure 1). 

The basic physicochemical properties of soil under different plant communities showed significant differences (*p* < 0.05) for all soil properties except pH and available N. Among them, soil nutrient contents and SOM were the highest in NSF, and soil physicochemical properties in PF were poor, with SOM, TP, and SWC being the lowest among the four communities (Table 2).

### 3.2. Functional Characteristics of Plant Communities

**Figure 2:**
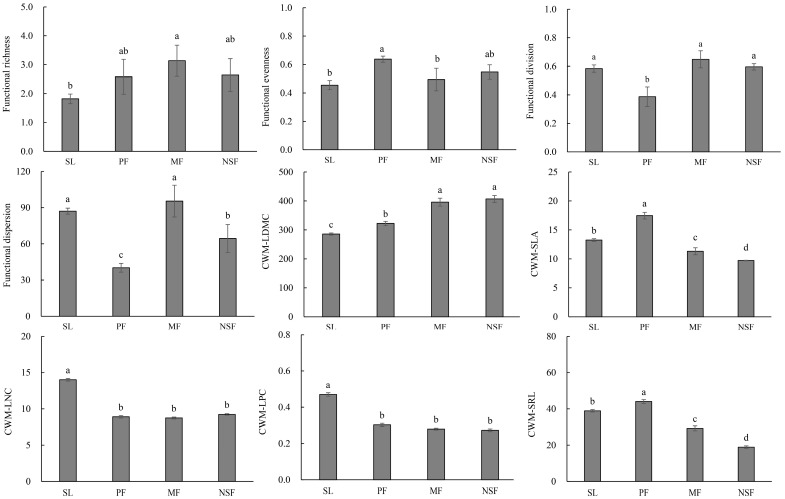
Functional characteristics (FD and CWMs) in four vegetation restoration types. Different letters indicate significant differences among the four restoration types at the 0.05 level.The CWM values of functional traits showed different distributions depending on the functional traits (Figure 2). CWM-LDMC tended to increase with the development of community structure complexity, with CWM-LDMC values in MF and NSF significantly higher than those in PF and SL. For SLA and SRL, the order of values of CWM was PF > SL > MF > NSF, with significant differences among all four communities. SL had significantly higher values of CWM-LNC and LPC than the other three communities, while there were no significant differences in CWM-LNC and LPC among PF, MF, and NSF (Figure 2).

The functional diversity in the four communities presented different distribution patterns depending on its parameters (Figure 2). The highest FRic was found in MF and the lowest in SL. A significant difference was found only between SL and MF. The values of FEve in PF were significantly higher than those in MF and SL. For FDiv and FDis, the four communities showed similar patterns. FDiv and FDis in PF were significantly lower than those in the other three plant communities (Figure 2).

### 3.3. Relationships among Functional Traits and Diversity Indices

At the species level, all traits were significantly correlated with each other. LDMC was negatively correlated with the other four traits, and SLA, LNC, LPC, and SRL showed positive linkages with each other. At the community level, the CWMs of five traits were strongly correlated with each other, except between CWM-LNC and SLA and SRL (Table 3).

Correlations between species diversity and FD showed that Shannon diversity and species evenness were significantly associated with FDiv and FDis. Moreover, Shannon diversity was also tightly correlated with CWM-LNC and LPC, and species evenness was strongly linked to CWM-SLA and LNC (Table 4). No significant correlations were found between species richness and indices of FD.

### 3.4. Influence of Functional Diversity

**Figure 3:**
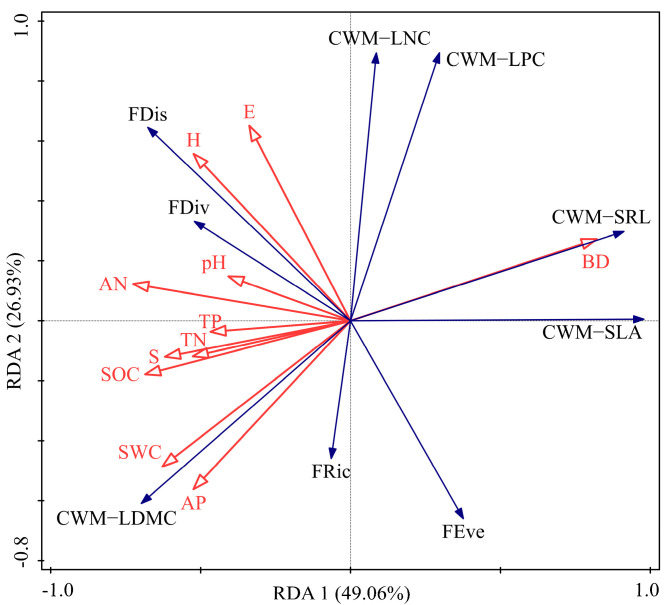
Redundancy analysis (RDA) of functional diversity indices and basic soil characteristics in four restoration communities. Functional diversity indices are represented as blue lines; soil characteristics and species diversity are represented as red lines.

Redundancy analysis (RDA) showed that the first two RDA axes explained a total of 75.98% of the variation in the different functional diversity indicators of the communities. Soil bulk density (BD) was correlated with CWM-SRL and CWM-SLA. A high correlation was also found between CWM-LDMC and SWC and AP (Figure 3). FDis and FDiv had close relationships to species diversity indicators (H, Shannon diversity, and E, species evenness). The results of correlation statistics showed that all the RDA axes of the functional attributes were closely linked to the species diversity and soil properties (SWC and AP) (Table 5).

The results of variation partitioning indicated that most of the variation in functional diversity across the restoration communities was explained by soil physicochemical properties. Specifically, the explanation rate from the soil properties was 46.42%, and that from species diversity was 12.12%. The joint explanation rate from soil properties and species diversity was 41.46%.

## 4. Discussion

### 4.1. Functional Characteristics among Vegetation Restoration Types

We found that different restoration communities altered most community structures and functional properties. FRic reflects the functional space occupied by a species [29]; thus, the relatively lower FRic value in SL suggests that some resource niches are empty and implies the potential migration risk of other species, including invasive species [10,30]. The higher FEve value in PF indicated that the effective utilization degree of some parts of niche space, while occupied, was higher than that in SL and MF [7,31]. The FDiv value in PF was significantly lower than that in the other three communities, indicating that PF has a low degree of niche differentiation or competition for soil resources [7]. The lower FDis of PF indicated a decrease in interpopulation or intrapopulation trait dispersion [32], implying that the habitat filter may play a role in community assembly [33]. Considerable changes were observed in the values of CWMs. This could be due to interspecific variation, intraspecific variation, or a combination of the two [12]. In general, the interspecific variability in the trait values of dominant species in communities was much greater than the intraspecific variability [12,34]. As a result, changes in CWMs may be attributed to the substitution of plant species with different functional traits.

### 4.2. Species Diversity and Functional Characteristics

We found significant correlations between functional traits at the species and community levels. In general, SLA, LNC, LPC, LDMC, and SRL are trait indicators of plant growth rate, and SLA, LNC, LPC, and SRL have positive correlations and negative correlations with LDMC [21,35]. The present study showed significant relationships among these five traits. Moreover, significant relationships among the CWMs of most traits were also found at the community level. These results indicated a potential covariation between these selected traits.

Significantly positive correlations were only found between FD (FDiv and Fdis) and species diversity (Shannon index and species evenness), indicating that the niche differentiation and interpopulation or intrapopulation trait dispersion significantly increased with Shannon diversity and species evenness [7]. These results indicated changes in the community assembly rule from environmental filtering in communities with low species diversity to ecological differentiation in communities with high species diversity [33]. However, no significant correlations were found between species diversity and FRic or FEve (Table 4). The weak linkage between species diversity and functional characteristics has been reported in some studies [36,37]. The main reason behind this phenomenon may be related to environmental stress and the community assembly rule. In general, functional characteristics increase slowly as species diversity increases in concert with increasing constraints on species assembly by environmental filtering [38]. In addition, changes in the CWMs of SLA, LNC, and LPC with species diversity may be due to the substitution of different dominant species and their abundance across communities [9,26].

### 4.3. Contributions of Soil Properties to Functional Characteristics 

The results of RDA and variation partitioning showed that most of the variation in functional characteristics (FD and CWM) across different restoration communities was explained by soil physicochemical properties, especially SWC and AP. The lower SWC in dry seasons and soil AP content became the key influencing factors affecting the growth of plant species and ecosystem production [39,40,41]. Moreover, the distribution of FD also suggested that habitat filters may function in community assembly [25]. These results indicated that soil moisture in the dry season and soil AP, as a habitat filter, regulated the species distribution and functional trait patterns, which in turn affected the functional characteristics of the restoration community types. Interestingly, we found that soil properties and species diversity cooperatively explained 41.46% of the variation in the functional attributes. Together with the significant linkage between species diversity and the RDA axes (Table 5), these findings imply that changes in functional diversity (FD and CWM) can be direct effects of species diversity and indirect effects of soil resources. 

In our study, we found that there were stronger linkages between CWMs and SWC and AP than FD (Figure 3), which implies that the dominant traits are more strongly determined by SWC and AP [12]. Many studies have reported that ecosystem functioning is primarily determined by the functional traits of the dominant species based on the mass ratio hypothesis [12,20,42,43]. In present study, there are obvious differences in biological attributers of the dominant species in each community. For example, dominant species in SL, PF, MF, and NSF belong to different life types, shrub, coniferous species, mixed needle–broad-leaved species, and broad-leaved species, respectively. Meanwhile, litter quality and quantity and mycorrhizal types also showed obvious difference in ecological and biological attributes [44,45,46]. Therefore, according to the results of this study, the selection of dominant species adapted to low soil moisture and available phosphorus and the construction of plant communities based on selected species can effectively drive community assembly and ecosystem functions in the vegetation restoration process. 

## 5. Conclusions

Our results suggest that different restoration communities altered most community structures and functional properties in terms of species diversity, FD, and CWM. The values of CWM and FD in different communities presented different distribution patterns depending on certain traits and parameters. Significant correlations among plant functional traits at the species and community scales indicated a potential covariation between these selected traits in communities. Weak correlations were found between functional characteristics and species diversity. Most of the variation in functional characteristics (FD and CWM) across different restoration communities was explained by soil physicochemical properties based on variation partitioning analysis. Redundancy analysis showed that soil physicochemical properties, especially SWC and AP, were the main factors in driving variation in functional characteristics. Moreover, the linkages between CWMs and soil properties were stronger than those between FD and soil properties. These results indicated that SWC and AP, as habitat filters, regulate the patterns of functional characteristics by changing the dominant species composition and functional traits of species. Therefore, the selection of restoration species adapted to low soil moisture and available phosphorus and the construction of plant communities based on selected species as the dominant species can effectively drive community assembly and ecosystem functions in the vegetation restoration process.

## Figures and Tables

**Table 1:** Species composition and structural attributes for four vegetation restoration types.

Type	Age (Year)	DBH (cm)	Tree Density (Trees/ha)	Cover	Common Dominant Species
SL	50–60	—	—	0.73 ± 0.16	*Ternstroemia gymnanthera*, *Eupatorium adenophorum*
PF	40–50	10.34 ± 2.68	2436 ± 1244	0.64 ± 0.15	*Pinus yunnanensis*
MF	40–50	9.77 ± 2.42	3946 ± 2803	0.83 ± 0.21	*P. yunnanensis*, *Keteleeria evelyniana*, *Cyclobalanopsis glaucoides*
NSF	10–20	7.43 ± 2.65	6612 ± 3733	0.85 ± 0.22	*K. evelyniana*, *C. glaucoides*, *Lithocarpus dealbatus*

Values are mean ± SD. SL, shrubland; PF, *Pinus yunnanensis* forest; MF, mixed coniferous and broad-leaved forest; NSF, natural secondary forest.

**Table 2:** Soil basic properties under four restoration community types.

Soil Index	SL	PF	MF	NSF
pH	4.25 ± 0.23 ^a^	4.16 ± 0.07 ^a^	4.32 ± 0.04 ^a^	4.28 ± 0.17 ^a^
BD (g/cm^3^)	1.25 ± 0.05 ^a^	1.34 ± 0.02 ^a^	1.14 ± 0.06 ^b^	0.98 ± 0.07 ^c^
SWC (%)	27.33 ± 6.23 ^ab^	25.36 ± 2.76 ^b^	34.88 ± 3.08 ^a^	38.45 ± 4.62 ^a^
TN (%)	0.08 ± 0.01 ^ab^	0.06 ± 0.02 ^ab^	0.05 ± 0.02 ^b^	0.10 ± 0.03 ^a^
AN (mg/kg)	81.01 ± 6.56 ^a^	54.74 ± 24.68 ^a^	59.71 ± 18.25 ^a^	107.81 ± 32.59 ^a^
TP (%)	0.02 ± 0.00 ^b^	0.01 ± 0.00 ^c^	0.02 ± 0.00 ^b^	0.03 ± 0.00 ^a^
AP (mg/kg)	0.64 ± 0.06 ^c^	1.35 ±0.53 ^ab^	1.04 ± 0.09 ^bc^	2.15 ± 1.15 ^a^
SOM (%)	2.03 ± 0.08 ^ab^	1.57 ± 0.58 ^b^	1.71 ± 0.10 ^ab^	2.75 ± 0.58 ^a^

Values are mean ± SD; dissimilar letters indicate significant differences among the four restoration types at the 0.05 level.

**Table 3:** Correlations among functional traits and among community-weighted means of traits for four restoration communities.

	LDMC	SLA	LNC	LPC	SRL
LDMC		−0.58 **	−0.71 **	−0.86 **	−0.64 **
SLA	−0.26 *		**0.31**	0.54 **	0.94 **
LNC	−0.44 **	0.50 **		0.74 **	**0.22**
LPC	−0.40 **	0.55 **	0.67 **		0.58 **
SRL	−0.34 **	0.37 **	0.47 **	0.42 **	

Bolded and non-bolded correlation coefficients represent the relationships between traits at the species level and the correlations between the CWMs of traits. Asterisks indicate a significant correlation (* *p* < 0.05 and ** *p* < 0.01).

**Table 4:** Relationships between species diversity and functional attributes for four restoration communities.

Functional Diversity	Species Diversity		
	Shannon	Richness	Evenness
FRic	0.09	0.44	−0.08
FEve	−0.16	0.01	−0.15
FDiv	0.53 **	−0.17	0.68 **
FDis	0.80 **	0.19	0.84 **
CWM-LDMC	−0.17	−0.24	−0.07
CWM-SLA	−0.37	0.13	−0.49 *
CWM-LNC	0.55 **	0.26	0.49 *
CWM-LPC	0.47 *	0.29	0.38
CWM-SRL	−0.10	0.18	−0.20

* *p* < 0.05 and ** *p* < 0.01.

**Table 5:** Correlation between the redundancy analysis axes (RDA) of functional indices (FD and CWM) and influencing factors (soil properties and species diversity).

	RDA1	RDA2	R^2^	*P*
H	0.21	−0.98	0.67	0.01 **
S	0.89	−0.45	0.61	0.01 **
E	−0.10	−1.00	0.54	0.04 *
pH	0.63	−0.78	0.19	0.38
SWC	0.99	0.16	0.59	0.02 *
BD	−0.99	0.11	0.45	0.05
SOM	1.00	−0.09	0.44	0.05
TN	0.99	0.17	0.04	0.84
TP	0.97	0.22	0.07	0.72
AN	0.69	−0.72	0.15	0.49
AP	0.88	0.47	0.56	0.02 *

R^2^ indicates the proportion of variance explained. * and ** denote correlations that are significant at the 0.05 and 0.01 levels based on the Monte Carlo permutation test (*n* = 999).

## Data Availability

Not applicable.
